# β-Eudesmol, an oxygenized sesquiterpene, stimulates appetite via TRPA1 and the autonomic nervous system

**DOI:** 10.1038/s41598-017-16150-6

**Published:** 2017-11-17

**Authors:** Kazuaki Ohara, Takafumi Fukuda, Yuko Ishida, Chika Takahashi, Rena Ohya, Mikio Katayama, Kunitoshi Uchida, Makoto Tominaga, Katsuya Nagai

**Affiliations:** 1 Research Laboratories for Health Science and Food Technologies, Kirin Company, Limited, Yokohama, Kanagawa 236-0004 Japan; 2 Division of Cell Signaling, Okazaki Institute for Integrative Bioscience (National Institute for Physiological Sciences), National Institute of Natural Sciences, Okazaki, Aichi 444-8787 Japan; 3 ANBAS Corporation, Osaka, 531-0072 Japan; 4 Present Address: Central Laboratories for Key Technologies, Kirin Company, Limited., Yokohama, Kanagawa 236-0004 Japan; 5 Present Address: Department of Physiological Science and Molecular Biology, Fukuoka Dental College, Fukuoka, 814-0193 Japan

## Abstract

Transient receptor potential ankyrin 1 (TRPA1) is a calcium-permeable non-selective cation channel, which is activated by various noxious or irritant substances in nature. TRPA1 activators have been generally recognized as noxious, however, foods and beverages containing TRPA1 activators are preferably consumed; the reasons for this discrepancy are not well understood. We demonstrate that TRPA1 is involved in the stimulatory appetite control mechanism. β-Eudesmol is an oxygenated sesquiterpene contained in medicinal or edible plants which activates TRPA1. Oral administration of β-eudesmol brought significant increments in food intake in rats and elevated plasma ghrelin levels. Gastric vagal nerve activity (GVNA) has been reported to affect feeding behavior. *In vivo* electrophysiological measurement of GVNA revealed that oral-ingestion of β-eudesmol significantly increased GVNA. This GVNA elevation was eliminated by TRPA1 inhibitor (HC-030031) treatment prior to β-eudesmol administration. The physiological effects of β-eudesmol, for example, incremental increase in food intake, ghrelin elevation and activation of GVNA, were significantly reduced in TRPA1 knockout rats. Our results indicated that β-eudesmol stimulates an increase in appetite through TRPA1, and suggests why TRPA1 activator containing foods and beverages are preferably consumed.

## Introduction

Transient receptor potential ankyrin 1 (TRPA1) is a calcium-permeable, non-selective, cation channel which is mainly expressed in sensory nerve cells. It is reported to play an important role in the susceptibility to various noxious stimuli, such as chemical, mechanical, and cold stimulus^1^ and pain sensitivity to inflammation^2^. TRPA1 antagonist development is considered to be important in the pharmaceutical industry. Agonists of TRPA1 are a treatment possibility for atonic and spastic motor stasis of the gut^3^, however, their use has been complicated since TRPA1 activators are generally nociceptive and cause pain or pungency at high doses^4–6^. Previous reports have focused on TRPA1 in cells other than sensory nerves and its various physiological roles have attracted much attention^7^.

Plants produce a variety of secondary metabolites which contribute to human life in the form of natural medicines, fragrances, seasoners and spices. Many plant secondary metabolites have been reported to activate TRPA1, for example, allyl isothiocyanate in horseradish is a TRPA1 activator^8^. In addition, compounds contained in the essential oils of plants are reported to be TRPA1 activators. The major components of essential oils are volatile terpenoids: menthol, contained in mint, and carvacrol, which accumulates in oregano, are reported to be activators of TRPA1^9,10^. These TRPA1 activators have been empirically preferred as food and beverage ingredients, despite excess amounts being a noxious stimulus. The physiological benefits of these TRPA1 activators in foods and beverages have not been well researched.

It was recently reported that β-eudesmol, an oxygenated sesquiterpene, could activate human TRPA1; the EC_50_ was determined to be 32.5 ± 0.38 μM^11^. β-Eudesmol has been found in medicinal plants and physiological effects on mammals have been reported^12–15^: β-eudesmol has been shown to inhibit the nicotinic acetylcholine receptor at 100–200 μM^13^. β-Eudesmol also accumulates in edible plants, especially a particular hop cultivar, and it has been reported to contribute to the spicy sensation of beer^16^. In this report, we reveal that oral-intragastric administration of β-eudesmol increases gastric vagal nerve activity (GVNA), which controls appetite and digestion. The suppression of GVNA inhibited nutrient digestion and absorption^17^, while activation of GVNA promoted nutrient digestion and absorption^18^. Oral administration of β-eudesmol affected the feeding behavior of rats and the appetite promoting effects of β-eudesmol were significantly reduced in TRPA1 knockout rats, which were established in this study. We also identified other TRPA1 activators which elevated GVNA. Our results provide scientific evidence to explain the mechanism of preferable intake of TRPA1 activators in foods and beverages.

## Results

### β-Eudesmol increased food intake and plasma ghrelin levels

**Figure 1:**
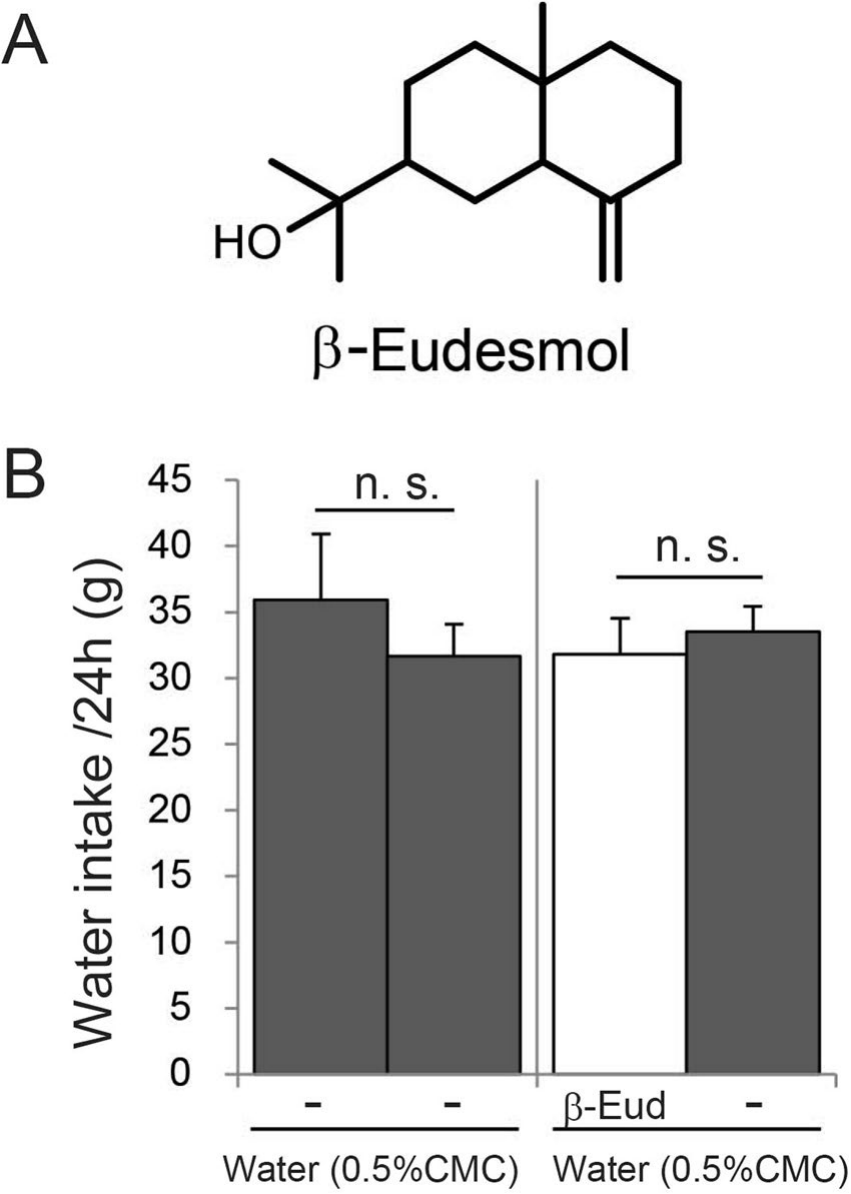
A two-bottle selection test for β-eudesmol in rats. (**A**) Structure of β-eudesmol. (**B**) Result of a two-bottle selection test. In the control experiment, water plus 0.5% CMC (control water) was supplied to both bottles (left). β-Eudesmol (5 ppb) containing 0.5% CMC water and control water were used in the next test (right). Values are means ± SEM (n = 15). Statistical differences were analyzed using the Mann-Whitney U test. CMC, carboxymethylcellulose; β-EUD, β-eudesmol; n. s., not significant.

**Figure 2:**
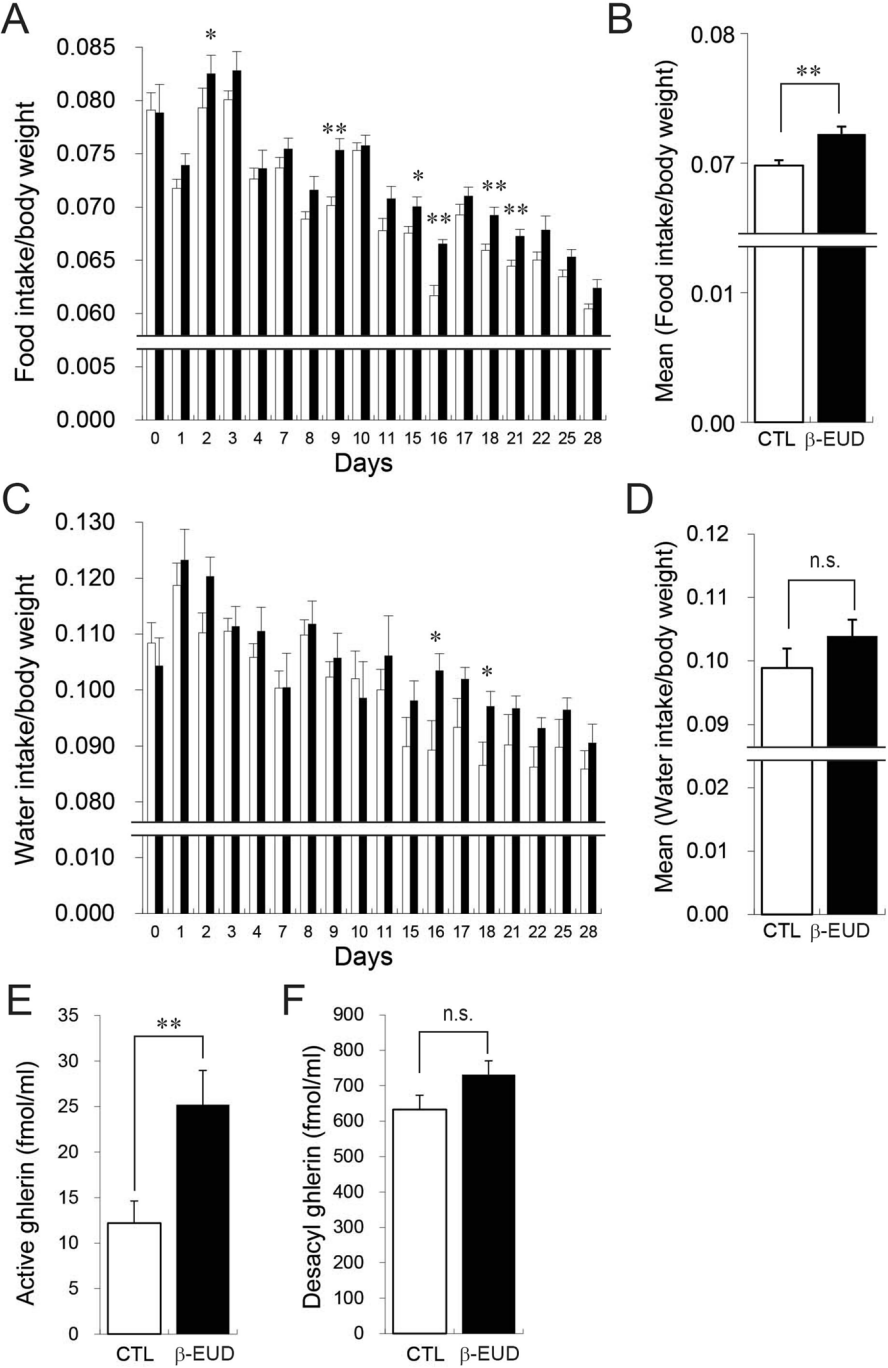
β-eudesmol increased food intake and plasma ghrelin. (**A**) Food intake in rats given β-eudesmol (0.14 ppb) containing 0.5% carboxymethylcellulose (CMC) water (n = 20). Food intake in the control group given 0.5% CMC containing water is shown by the open bar in the graph (n = 20). (**B**) Mean food intake during the experimental periods. (**C**) Water intake of rats given β-eudesmol (0.14 ppb) containing 0.5% CMC water. The control is shown by the white bar in the graph. (**D**) Mean water intake during experimental periods. (**E**) Plasma octanoyl ghrelin levels in β-eudesmol administered rats. (**F**) Plasma desacyl ghrelin levels in β-eudesmol administered rats. Values are means ± SEM. Statistical differences were analyzed by the Mann-Whitney U test. *p < 0.05; **p < 0.01. CTL, control; β -EUD, β -eudesmol.

Aqueous solutions of β-eudesmol have no color, and the odor threshold of β-eudesmol has been reported to be 10000 ppb^16^. To determine whether rats could distinguish between β-eudesmol-containing water and control water, a two-bottle selection test was performed using β-eudesmol at 5 ppb. Due to the hydrophobic nature of β-eudesmol, 0.5% carboxymethylcellulose was used as a dispersant. Control water contained 0.5% carboxymethylcellulose only. The results showed that the rats could not distinguish between water containing β-eudesmol and control water (Fig. 1). We then investigated the effects of β-eudesmol on feeding behavior. Wild type rats were given water containing β-eudesmol throughout the experimental period. The β-eudesmol concentration in water was almost the same level as that in beer (0.14 ppb), and did not cause drink avoidance behavior. Rats were divided into two groups, matched for food intake during the assimilation period, with different drinking water: water containing β-eudesmol and 0.5% carboxymethylcellulose or control water containing 0.5% carboxymethylcellulose only. Average of daily food intake increased in β-eudesmol group during the study period (between 100.6 to 107.9%, in comparison to control group) except for day 0. In the study period, a significant increase in food intake was observed in β-eudesmol group on days 2, 9, 15, 16, 18 and 21 (Fig. 2A). Food intake of whole study period in the β-eudesmol group was significantly higher than in the control group (103.5%, Fig. 2B). Water intake between the two groups was not significantly different, except on days 16 and 18 when it was significantly increased in the β-eudesmol group (Fig. 2C,D). These results showed that β-eudesmol brought modest but significant food intake increase in rat. There were no significant differences in body and major organ weights between the groups (Table 1). In accordance with food intake increment, plasma octanoyl ghrelin levels were significantly increased, while desacyl ghrelin levels were not affected (Fig. 2E,F). These results suggest that oral-administration of β-eudesmol modulates feeding behavior in rat.

**Table 1:** The effects of β-eudesmol on body and organ weights in wild type rats.

Wild type rats	Control (n = 20)	β-Eudesmol (n = 20)
Initial body weight (g)	288.2 ± 3.00	281.4 ± 4.44
Final body weight (g)	368.1 ± 5.49	358.0 ± 5.44
Liver (g)	10.4 ± 0.19	10.1 ± 0.23
Spleen (g)	0.60 ± 0.01	0.57 ± 0.02
Epididymal fat weight (g)	4.44 ± 0.15	4.71 ± 0.18
Retroperitoneal fat weight (g)	6.00 ± 0.28	6.00 ± 0.33

No significant differences were observed between the control and β-eudesmol administered groups using the Mann-Whitney U test. Values are presented as the mean ± s.d.

### β-Eudesmol modulates gastric vagal nerve activity

**Figure 3:**
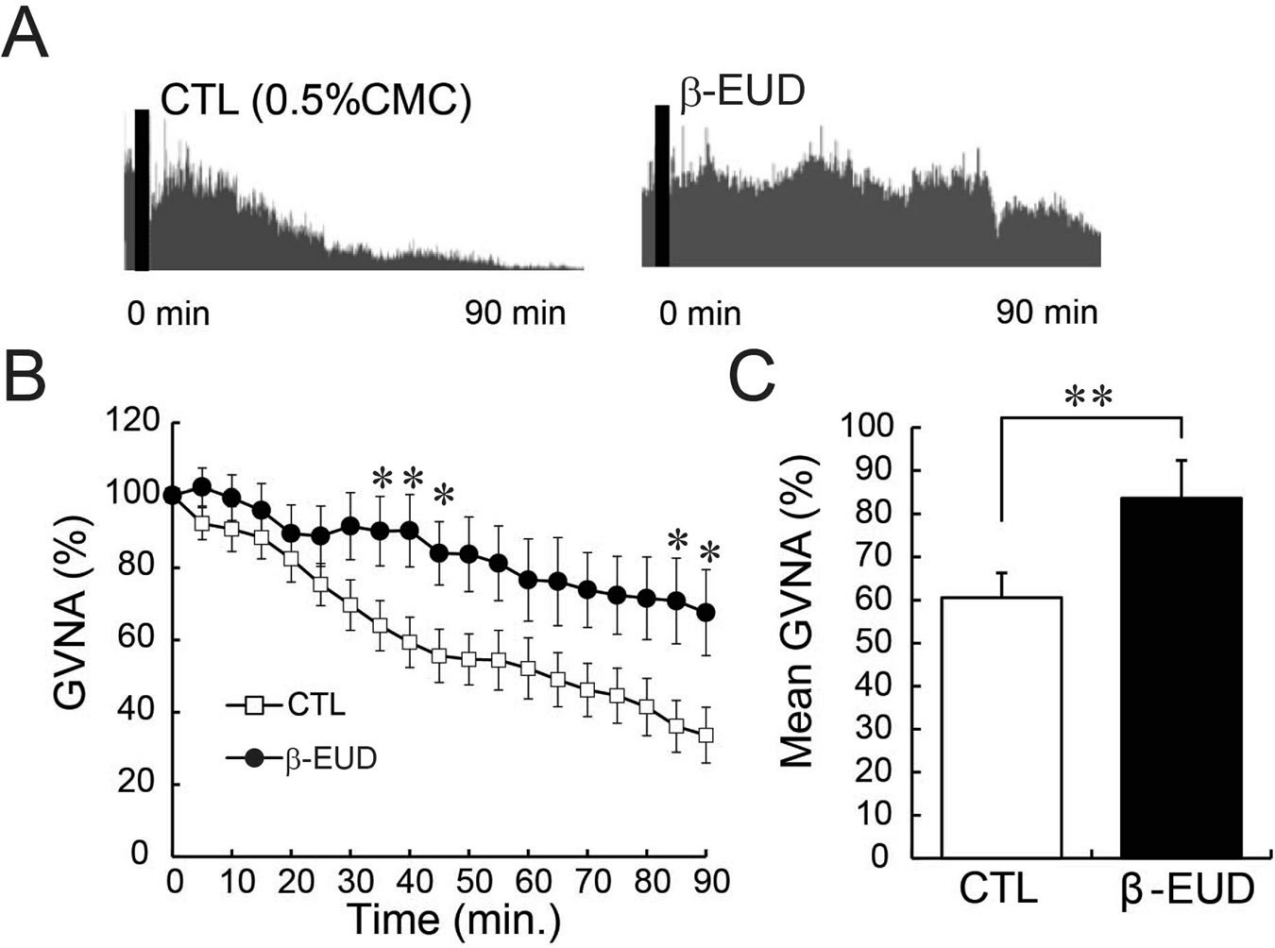
β-Eudesmol modulated gastric vagal nerve activity (GVNA). (**A**) Representative recordings of GVNA in rats administered 0.5% carboxymethylcellulose (CMC) or β-eudesmol (5 ppb) containing 0.5% CMC water. (**B**,**C**) Effect of β-eudesmol administration on GVNA (n = 9). Values are means ± SEM. Statistical differences were analyzed by the Mann-Whitney U test. *p < 0.05; **p < 0.01. CTL, control; β-EUD, β -eudesmol.

**Figure 4:**
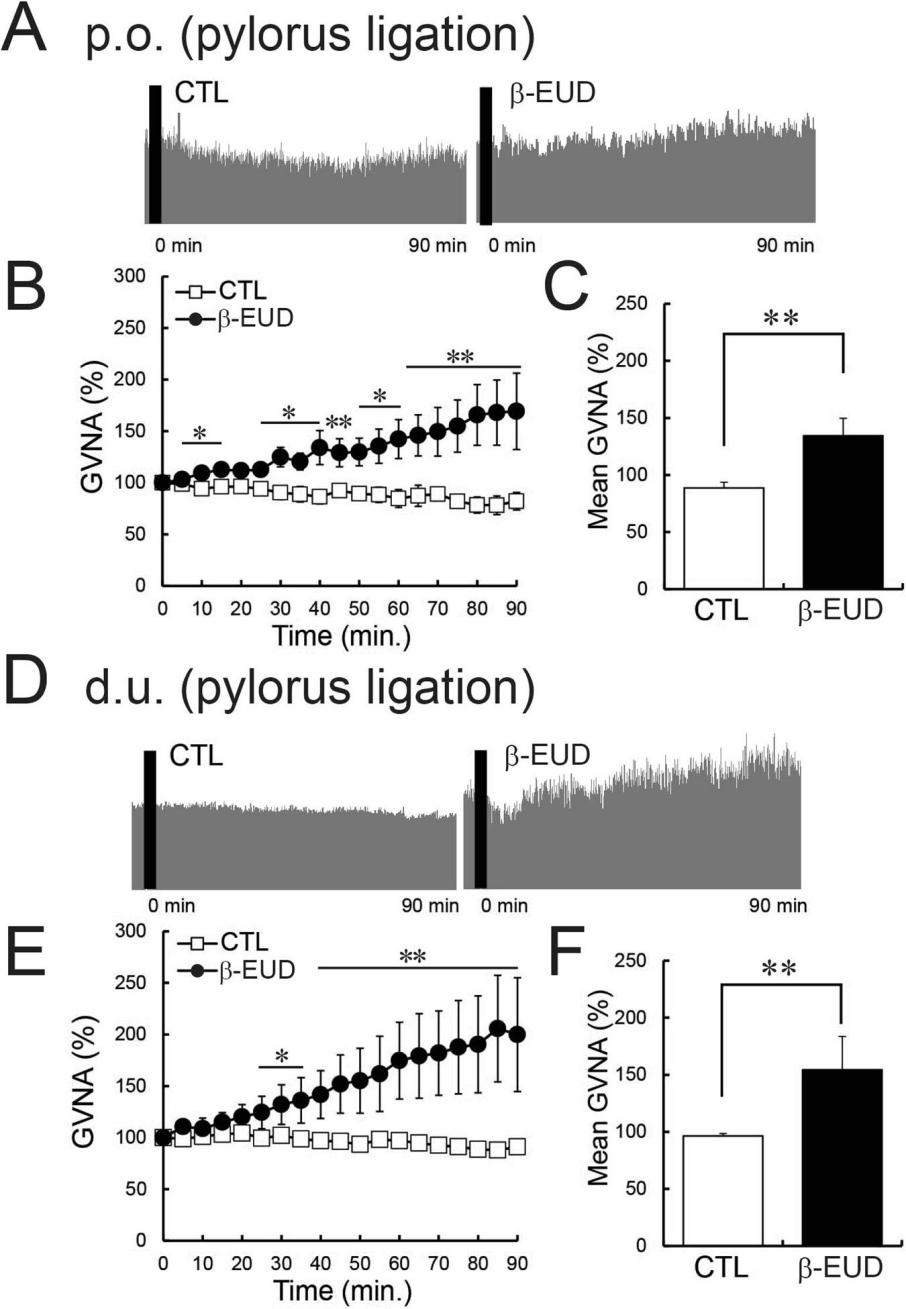
Effects of the β-eudesmol administration route on GVNA. (**A**) Representative recordings of GVNA in pylorus ligated rats administered 0.5% carboxymethylcellulose (CMC) or β-eudesmol (5 ppb) containing 0.5% CMC water by oral administration (p.o.). (**B**,**C**) Effects of oral administration (p.o.) of β-eudesmol in pylorus ligated rats (n = 5). (**D**) Representative recordings of GVNA in pylorus ligated rats administered 0.5% carboxymethylcellulose (CMC) or β-eudesmol (5 ppb) containing 0.5% CMC water by intraduodenal administration (d.u.). (**E**,**F**) Effects intraduodenal administration (d.u.) of β-eudesmol in pylorus ligated rats (n = 5). Values are means ± SEM. Statistical differences were analyzed by the Mann-Whitney U test. *p < 0.05; **p < 0.01. CTL, control; β-EUD, β -eudesmol.

**Figure 5:**
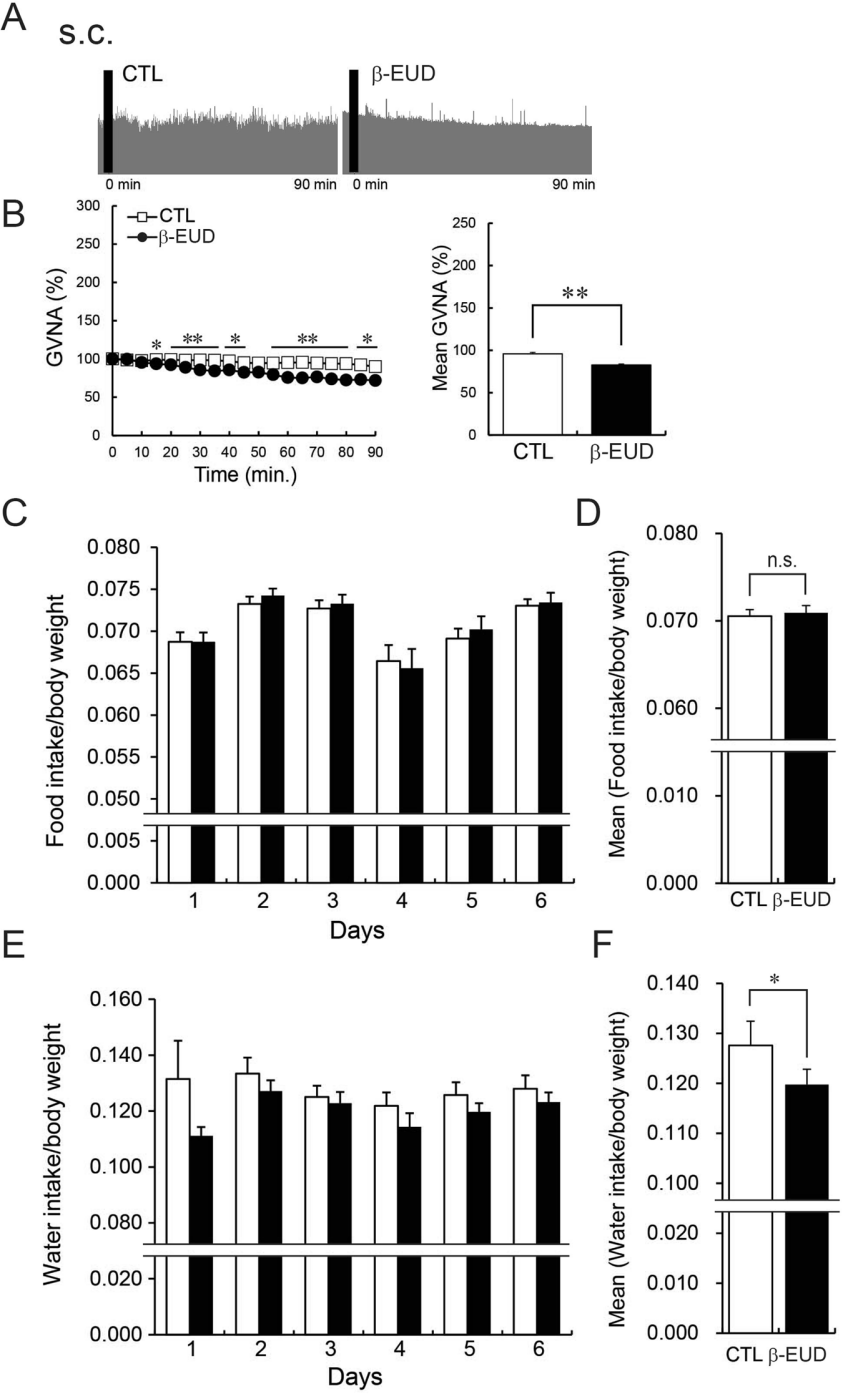
The effect of subcutaneous administration of β-eudesmol on GVNA, food and water intake. (**A**) Representative recordings of GVNA in pylorus ligated rats administered 0.5% carboxymethylcellulose (CMC) or β-eudesmol (5 ppb) containing 0.5% CMC water by subcutaneous administration (s.c.). (**B**) Effects of subcutaneous administration (s.c.) of β-eudesmol on GVNA in rats (n = 5). (**C**) Food intake in rats given β-eudesmol (n = 10). Food intake in the control group is shown by an open bar in the graph (n = 10). (**D**) Mean food intake during experimental periods. (**E**) Water intake in rats given β-eudesmol. The control is shown by a white bar in the graph. (**F**) Mean water intake during experimental periods. Values are means ± SEM. Statistical differences were analyzed using the Mann-Whitney U test. *p < 0.05. n. s., not significant. CTL, control; β-EUD, β -eudesmol.

To reveal the mechanisms underlying appetite stimulation by β-eudesmol, gastric vagal nerve activity (GVNA) was measured. GVNA elevation has been reported to increase food intake^18^. *In vivo* electrophysiological experiments showed that a gradual decrease in GVNA was observed with administration of a control solution (0.5% carboxymethylcellulose), in comparison, β-eudesmol intragastric administration significantly enhanced GVNA (Fig. 3). GVNA elevation after intragastric administration and intraduodenal administration of β-eudesmol was also observed in pylorus ligated rats (Fig. 4A–C and D–F, respectively). Subcutaneous administration of β-eudesmol significantly decreased GVNA (Fig. 5A,B). Consistent with this result, water intake after subcutaneous administration of β-eudesmol was significantly decreased, however, food intake was not affected (Fig. 5C–F). Previous studies showed that the effects of GVNA upregulation, such as increasing food intake, were greater than the downregulated effects^17,18^. This might explain why we only detected a decrease in water intake after subcutaneous administration of β-eudesmol. These results suggested that the digestive tract may be the target organ contributing towards GVNA elevation by β-eudesmol.

**Figure 6:**
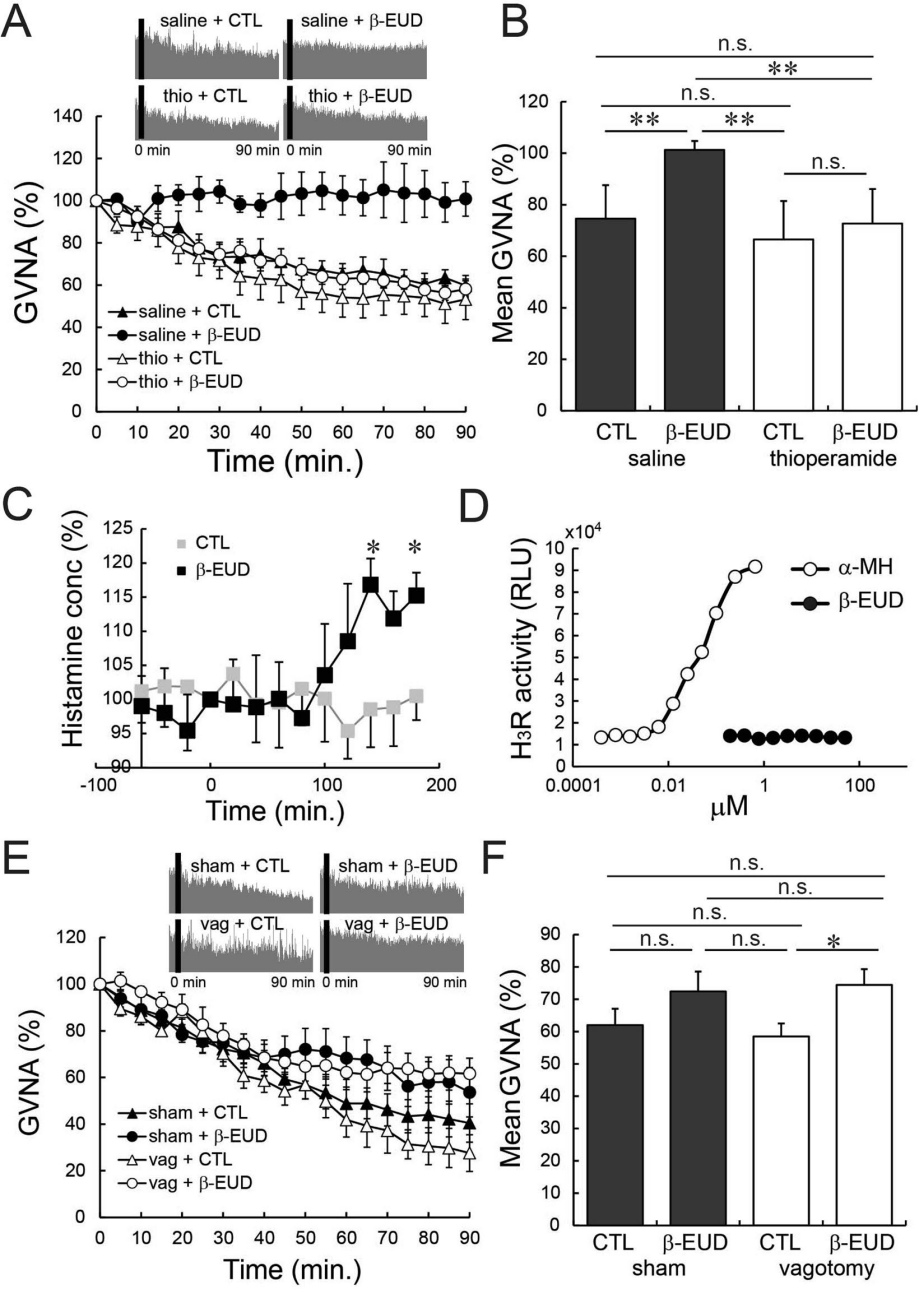
β-Eudesmol derived GVNA elevation involves the histamine H_3_ receptor. (**A**,**B**) Changes in β-eudesmol derived GVNA in rats previously administered thioperamide (n = 5). (**C**) Histamine concentration in the tuberomammillary nucleus in β-eudesmol administered rats (n = 5). The histamine concentration at 100% was 17.5 fmol/20 μl (CTL) and 19.1 fmol/20 μl (β-EUD). (**D**) *In vitro* histamine H_3_ receptor activity measurements. (**E**,**F**) The effects of subdiaphragmatic vagotomy on β-eudesmol derived GVNA elevation (n = 5). Statistical differences were analyzed by the Mann-Whitney U test or the Kruskal Wallis test followed by Steel-Dwass. *p < 0.05; **p < 0.01. CTL, control; β-EUD, β -eudesmol; n. s., not significant; α-MH, α-methylhistamine.

Efferent parasympathetic nerve activity elevation was reported to be under the control of the histamine H_3_ receptor in the autonomic nerve center^18^. In this study, GVNA enhancement by β-eudesmol was completely eliminated by prior administration of a histamine H_3_ receptor antagonist, thioperamide (Fig. 6A,B). In addition, microdialysis experiments showed that intragastric administration of β-eudesmol produced a significant elevation in extracellular histamine concentration in the tuberomammillary nucleus (TMN) of the hypothalamus, which is thought to be the autonomic center (Fig. 6C). These results suggested that GVNA elevation by β-eudesmol may involve histamine release in synapses and histamine H_3_ receptor activation in the autonomic center. We also confirmed that β-eudesmol did not directly activate the histamine H_3_ receptor *in vitro* (Fig. 6D), suggesting that β-eudesmol indirectly affected histamine release in the autonomic center. We adapted the subdiaphragmatic vagotomy technique to confirm whether afferent nerve activity from the gastrointestinal tract to the autonomic center was involved in β-eudesmol derived GVNA elevation. The results demonstrated that β-eudesmol derived GVNA elevation was not eliminated in vagotomized rats (Fig. 6E,F), suggesting afferent autonomic nerve activity from the gastrointestinal tract has little effect on β-eudesmol derived GVNA elevation.

### β-Eudesmol activated rat TRPA1

**Figure 7:**
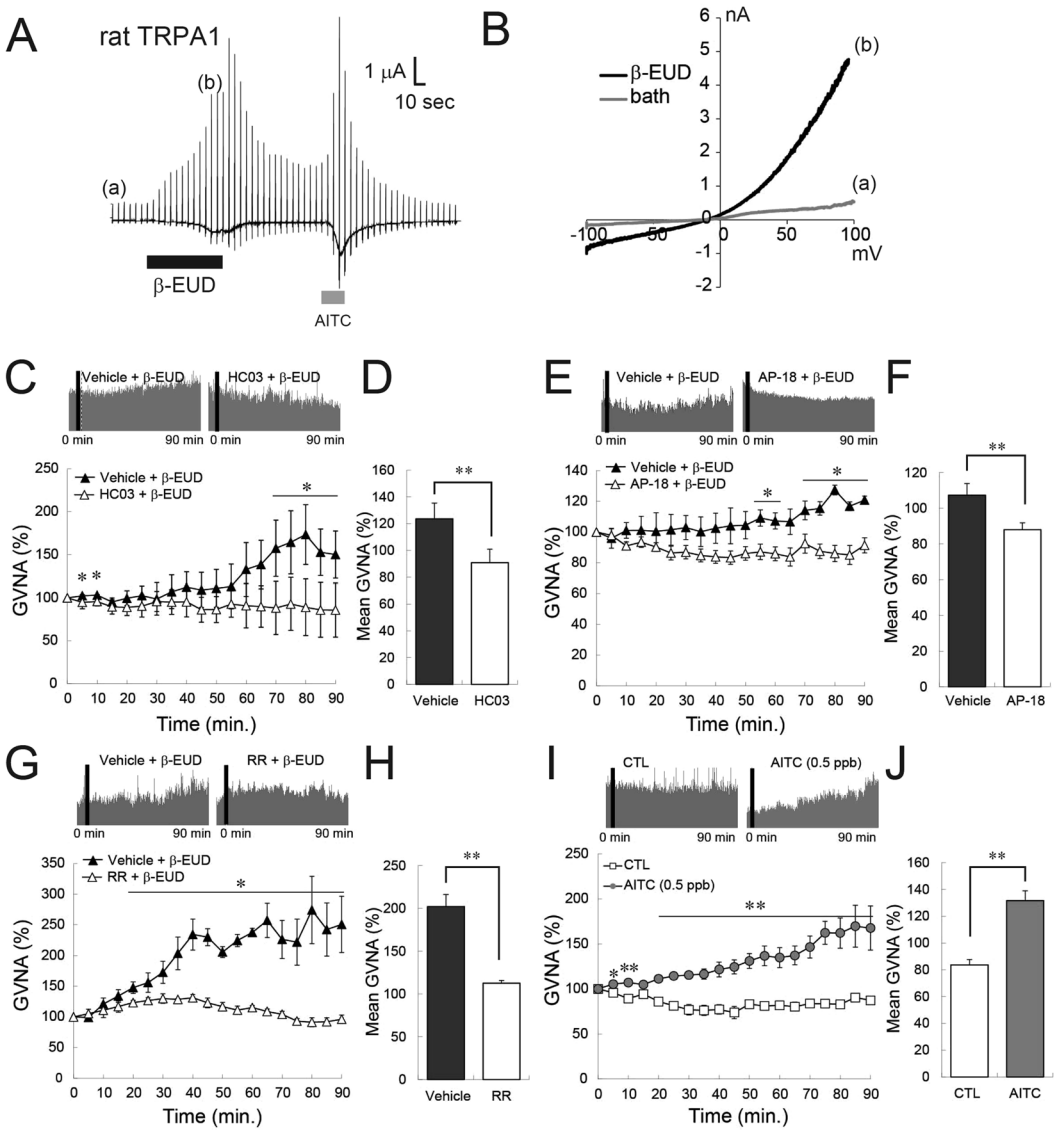
TRPA1 inhibitors and activators affected GVNA in rats and β-eudesmol activated rat TRPA1. (**A**) Representative traces of whole-cell patch-clamp currents activated by β-eudesmol (60 μM) in HEK293 cells expressing rat TRPA1. AITC (100 μM) was used as a TRPA1 stimulant. (a), (b) Indicate the points of ramp pulse application to generate curves I–V shown in (**B**,**C**,**D**). The effect of a selective TRPA1 inhibitor, HC-030031 (300 mg/kg), on β-eudesmol derived GVNA elevation (n = 3). (**E**,**F**). The effect of AP-18 (1 mg/kg), a selective TRPA1 inhibitor, on β-eudesmol derived GVNA elevation (n = 3). (**G**,**H**). The effect of a non-selective TRPA1 inhibitor, Ruthenium Red (1 mg/kg), on β-eudesmol derived GVNA elevation (n = 3). (**I**,**J**). The effect of a representative TRPA1 activator, AITC, on GVNA (n = 5). Values are means ± SEM. Statistical differences were analyzed by the Mann-Whitney U test. *p < 0.05; **p < 0.01. AITC, allyl isothiocyanate; CTL, control; β-EUD, β-eudesmol; HC03, HC-030031; RR, Ruthenium Red.

β-Eudesmol was reported to activate the human TRPA1 channel, however, interspecies differences have been reported by other investigations among TRPA1 activators^19,20^. We carried out electrophysiological experiments to confirm that β-eudesmol activates TRPA1, using HEK293 cells transiently expressing rat TRPA1. Whole cell patch clamp experiments showed that β-eudesmol brought gradual inward currents with an outwardly rectifying current-voltage relationship, a characteristic property of the TRPA1 channel (Fig. 7A,B). These results confirmed that β-eudesmol activated rat TRPA1.

### TRPA1 activators modulate gastric vagal nerve activity

To reveal the involvement of TRPA1 channels in β-eudesmol derived GVNA enhancement, a selective TRPA1 inhibitor (HC-030031) was used in wild type rats. Oral administration of HC-030031 (300 mg/kg) prior to β-eudesmol administration significantly reduced β-eudesmol derived GVNA enhancement (Fig. 7C,D). An additional selective TRPA1 inhibitor, AP-18 (1 mg/kg), and a non-selective TRPA1 inhibitor, ruthenium red (1 mg/kg), also significantly reduced GVNA enhancement by β-eudesmol (Fig. 7E–H).

In addition to β-eudesmol, other TRPA1 activators are found as food ingredients, for example, AITC in horseradish. To confirm whether GVNA can be enhanced by other TRPA1 activators, GVNA was measured in rats after oral administration of AITC. We found that AITC also significantly enhanced GVNA (Fig. 7I,J), suggesting that TRPA1 is just one of other candidate molecules capable of activating GVNA and affecting feeding behavior.

### TRPA1 knockout phenotype did not affect spontaneous feeding behavior in rats

**Figure 8:**
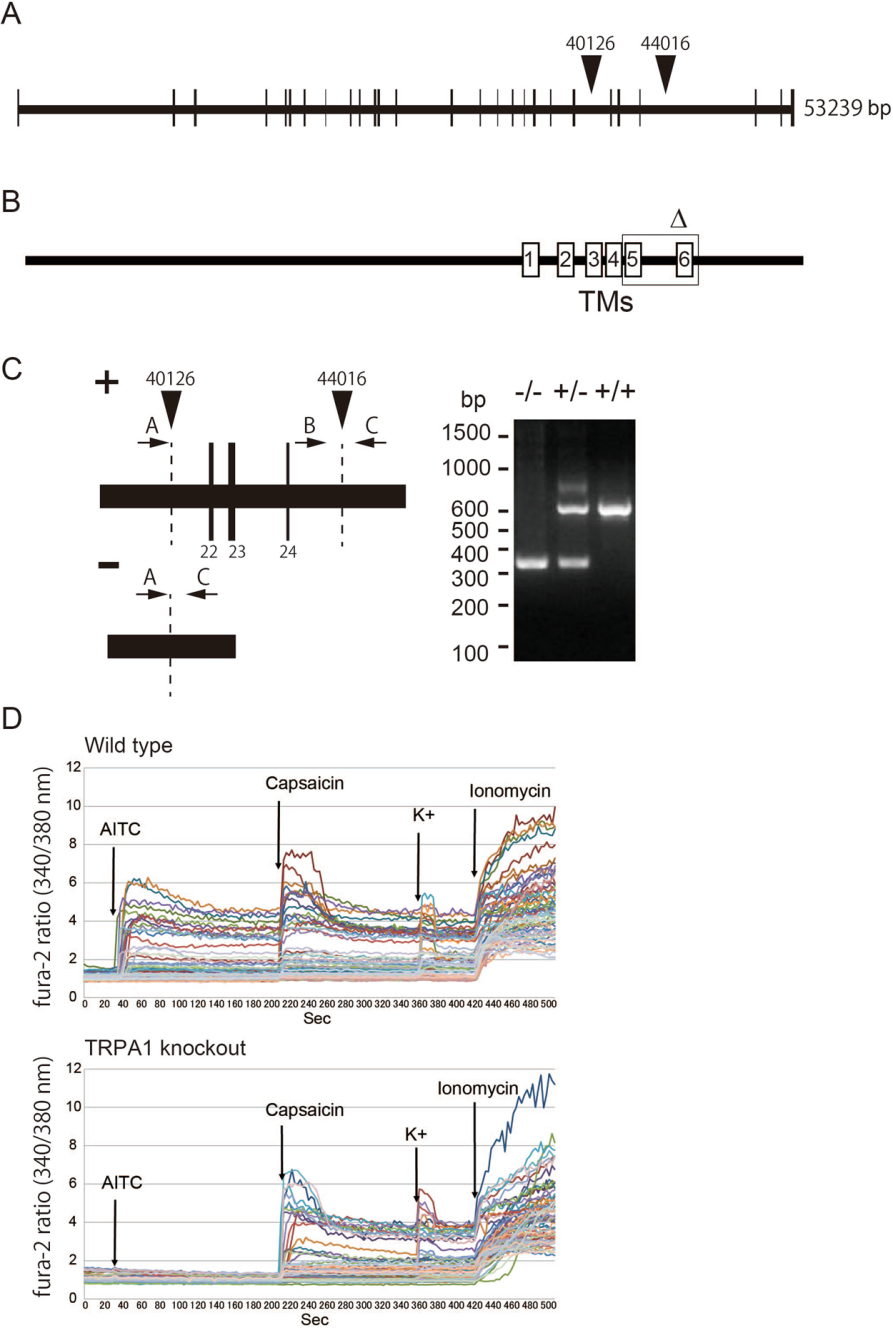
Generation of *TRPA1* knockout rats. (**A**) Exon-intron structure of rat *TRPA1* gene. The nucleotide sequence between the two arrow heads was deleted using zinc finger nuclease. (**B**) Transmembrane domain of the TRPA1 protein. The region enclosed by the box (Δ) was deleted. The deleted region contains the pore domain between S5 and S6. (**C**) Representative result of genotyping wild-type, heterozygote and TRPA1 knockout rats using genomic PCR and the primer set described in Materials and Methods. (**D**) Calcium imaging experiments of dorsal root ganglion neurons from wild type or TRPA1 knockout rats.

The increase in GVNA levels in rats after treatment with TRPA1 activators suggested that TRPA1 may be involved in feeding behavior. To confirm this, TRPA1 knockout rats were generated by zinc finger nuclease technology^21^: the genomic sequence between exons 22 and 24, which encode an ion channel pore region in rat TRPA1, was deleted. TRPA1 knockout was confirmed by sequencing the *TRPA1* gene, genomic PCR, and calcium imaging of dorsal root ganglion, as shown in Fig. 8. There was no significant difference in food intake between wild type and TRPA1 knockout rats (Table 2), suggesting that TRPA1 may not be involved in spontaneous feeding behavior in rats.

**Table 2:** Comparison of body weight and food intake between wild type and TRPA1 knockout rats.

	Wild type (n = 10)	TRPA1 knockout (n = 10)
Body weight, 7 wks (g)	277.2 ± 18.4	278.5 ± 14.3
Body weight, 12 wks (g)	435.6 ± 26.9	442.4 ± 35.8
Total food intake (g/6wks)	745.1 ± 52.6	783.0 ± 69.2

No significant differences were observed in body weights and food intake between wild type and TRPA1 knockout rats using the Mann-Whitney U test. Values are presented as the mean ± s.d.

### TRPA1 contributes to β-eudesmol derived gastric vagal nerve activity elevation and food intake increment

**Figure 9:**
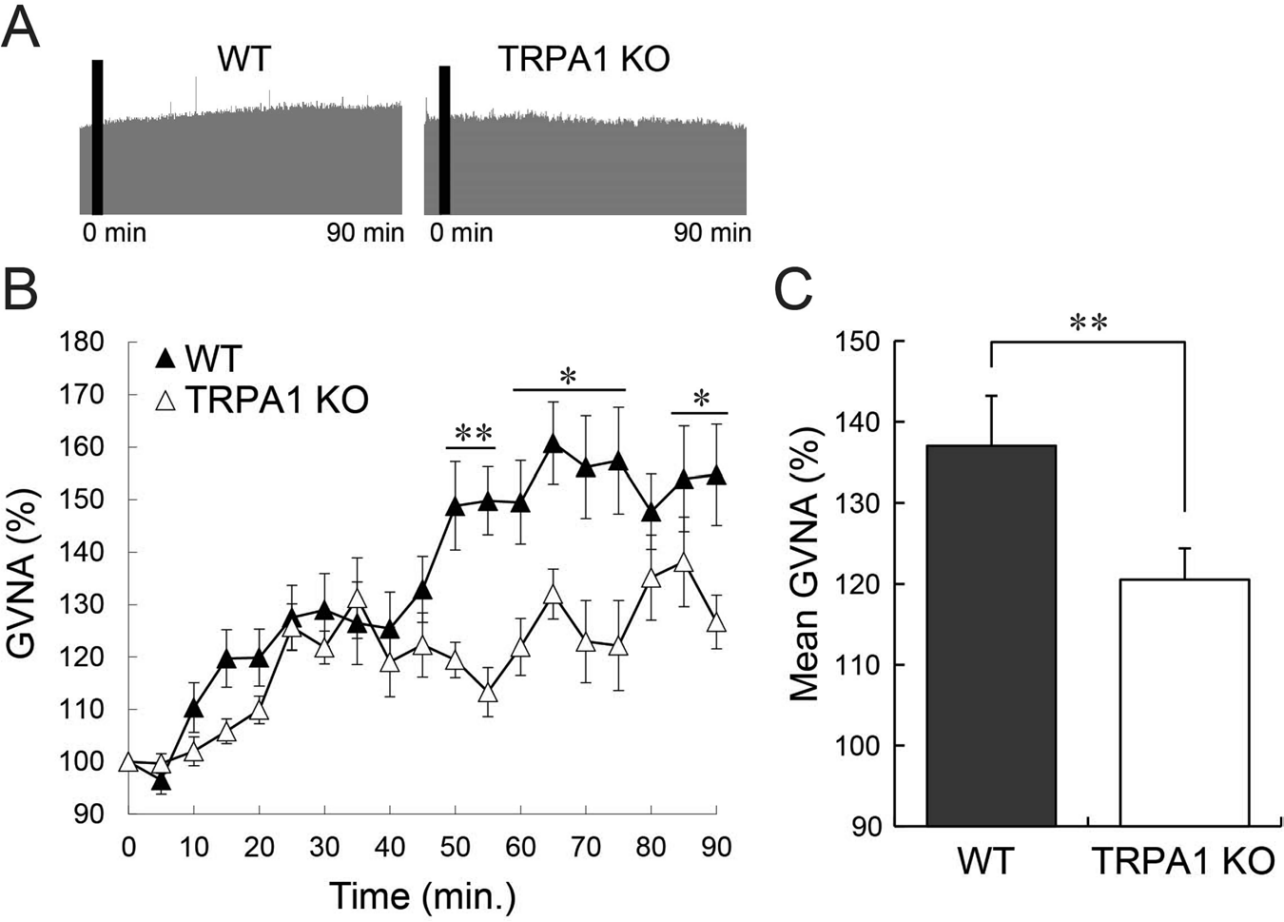
The effect of β-eudesmol on GVNA in TRPA1 knockout rats. (**A**) Representative recordings of GVNA in rats administered β-eudesmol (5 ppb) containing 0.5% CMC water. (**B**,**C**) The effect of β-eudesmol administration on GVNA (n = 6). Values are means ± SEM. Statistical differences were analyzed using the Mann-Whitney U test. *p < 0.05; **p < 0.01. CTL, control; β-EUD, β-eudesmol; WT, wild type.

**Figure 10:**
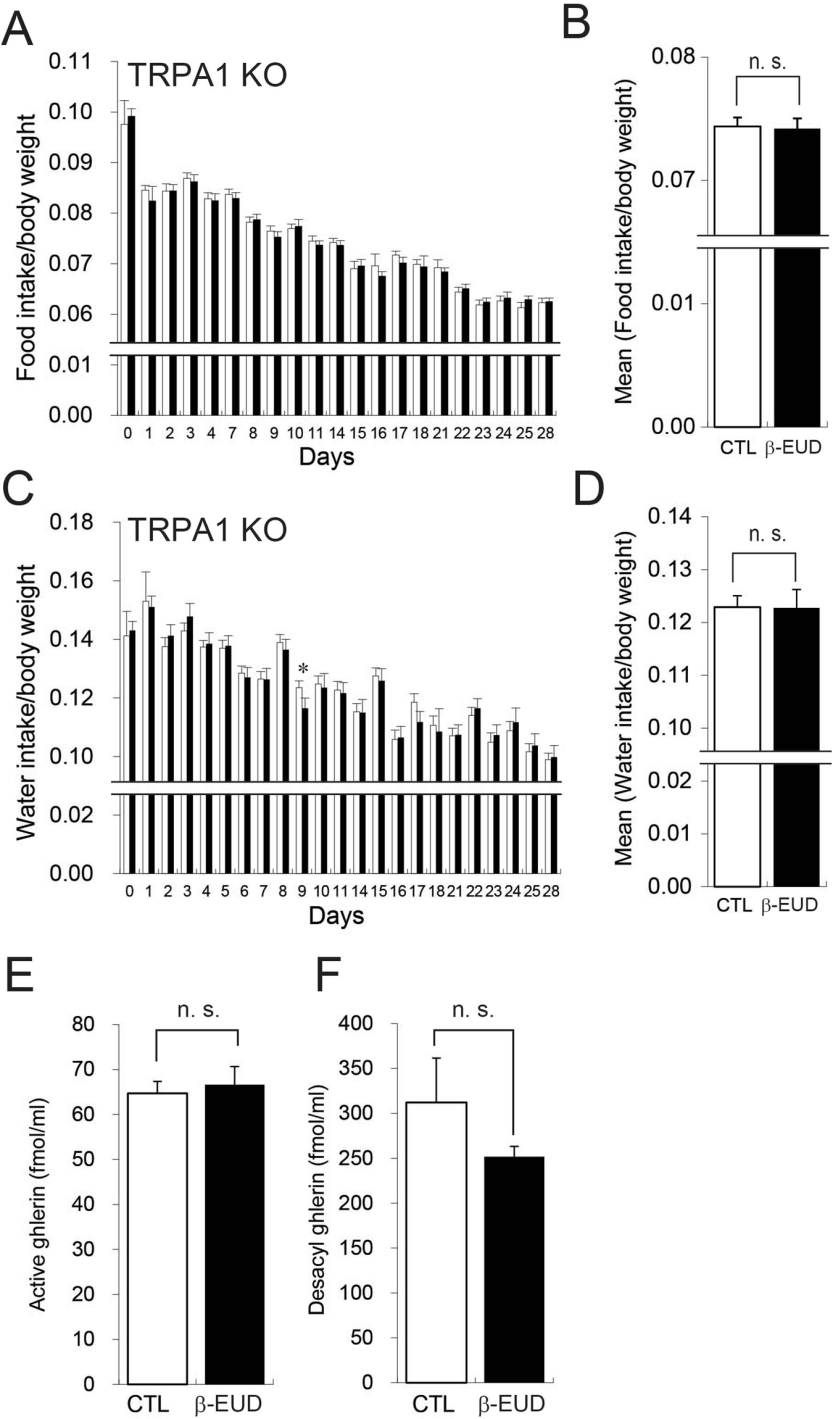
The effects of β-eudesmol on food intake and octanoyl ghrelin in TRPA1 knockout rats. (**A**) Food intake in rats given β-eudesmol (0.14 ppb) containing 0.5% carboxymethylcellulose (CMC) water (n = 17). Food intake in the control, 0.5% CMC water, is shown by a white bar in the graph (n = 19). (**B**) Mean food intake during experimental periods. (**C**) Water intake in rats given β-eudesmol (0.14 ppb) containing water. The control is shown by a white bar in the graph. (**B**) Mean water intake during experimental periods. (**E**) Plasma octanoyl ghrelin levels in β-eudesmol administered rats. (**F**) Plasma desacyl ghrelin levels in β-eudesmol administered rats. Values are means ± SEM. Statistical differences were analyzed using the Mann-Whitney U test. CTL, control; β -EUD, β-eudesmol; n. s., not significant.

TRPA1 knockout rats were utilized to confirm the involvement of TRPA1 in the increase in β-eudesmol derived feeding. GVNA enhancement by β-eudesmol was significantly decreased in TRPA1 knockout rats compared with wild type rats (Fig. 9), suggesting that TRPA1 channels are involved in β-eudesmol derived GVNA enhancement. In addition, there were no increases in β-eudesmol derived food intake and ghrelin levels in TRPA1 knockout rats (Fig. 10). There were no significant differences in body and major organs weights between the groups (Table 3). These results suggested that modulation of feeding behavior by β-eudesmol partly depended on TRPA1, and TRPA1 may contribute, not to spontaneous feeding behavior, but to mechanisms stimulating appetite control.

**Table 3:** The effects of β-eudesmol on body and organ weights in TRPA1 knockout rats.

TRPA1 knockout rats	Control (n = 19)	β-Eudesmol (n = 17)
Initial body weight (g)	290.7 ± 2.39	285.4 ± 2.64
Final body weight (g)	399.4 ± 5.32	390.1 ± 5.60
Liver (g)	11.6 ± 0.21	11.4 ± 0.21
Spleen (g)	0.68 ± 0.02	0.66 ± 0.02
Epididymal fat weight (g)	4.78 ± 0.22	4.82 ± 0.21
Retroperitoneal fat weight (g)	4.98 ± 0.32	5.11 ± 0.24

No significant differences were observed between the control and β-eudesmol administered groups using the Mann-Whitney U test. Values are presented as the mean ± s.d.

## Discussion

TRPA1 is reported to be a nociceptive receptor and its major physiological role is suggested to be a sensor to avoid noxious stimuli^1^. TRPA1 activators in nature are reported to be repellents^22,23^. Spices and seasoners containing TRPA1 activators have been preferably utilized in foods and beverages, and they are reported to stimulate the appetite^24^, however, the mechanisms underlying appetite stimulation have not been elucidated. Our research suggests that appetite stimulation by spices or seasoners may involve GVNA elevation, in part through TRPA1 activation.

In addition to β-eudesmol, other appetite stimulants have been previously reported. Ghrelin is the only known endogenous appetite-enhancing hormone to produce increases in food intake and body weight^25^. In our study, oral administration of β-eudesmol significantly increased blood ghrelin concentration and food intake of whole study period (by 3.5%), however, no significant increases in body or fat weight were observed. The increase in ghrelin concentration stimulated by β-eudesmol was in the order of several 10 fmol/ml, which may not be as high as the concentration previously reported to increase body weight (2.4–4.5 μmol/kg/day)^25^. In addition to stimulating an increase in food intake, ghrelin is known to lower lipid metabolism. Oral administration of a TRPA1 activator has been reported to increase energy expenditure^26,27^. We suggest that metabolic stimulation by β-eudesmol, a TRPA1 activating compound, may be the reason why weight gain does not accompany the increase in food intake caused by β-eudesmol.

Ghrelin does not produce a visible physiological response unless the route of administration is invasive. There are some examples in which an improvement effect was observed after oral-administration of natural compounds: bitter compounds transiently increased food intake by 120% via a bitter taste receptor, but they also suppressed digestion in the stomach, so that the long term food intake was decreased by 49%^28^. Our study showed that β-eudesmol had a continuous effect on increasing the appetite, which was measurable for at least one month. It was suggested that β -eudesmol may not affect the appetite via bitter taste receptors.

Rikkunshito, a herbal medicine, is known to have a beneficial effect on anorexia, a side effect of the anticancer drug, cisplatin. Rikkunshito increased the concentration of ghrelin through the inhibition of peripheral 5-HT_2B_ and central 5-HT_2C_ against cisplatin. In addition to increasing ghrelin secretion from the stomach, Rikkunshito also increased the reactivity of the ghrelin receptor through PGE2 inhibition^29^. In mice, the increase in ghrelin concentration after oral administration of Rikkunshito was about 10 fmol/ml, which is almost equivalent to the increase after β-eudesmol administration. Its appetite promoting effect was not transient but continuous, also similar to β-eudesmol^30^. Although an effect of restoring appetite during chemotherapy has been recognized, the effect of increasing food intake itself from baseline has not been reported. β-Eudesmol-mediated food intake stimulation via TRPA1 may suggest an alternative mechanism to the known appetite-stimulating agents. TRPA1 knockout rats showed normal food intake when compared with wild type rats (Table 2), suggesting that TRPA1 does not contribute to spontaneous appetite increases. Our results indicated that TRPA1 is likely to be involved in an appetite stimulation mechanism, caused by external stimuli from food and beverage ingredients.

Autonomic nerve activity, including GVNA, was reported to be totally controlled by histamine neurons in the autonomic center of the hypothalamus^31^. Our results suggested the target organ of β-eudesmol to generate GVNA elevation was the gastrointestinal tract. Thus, information generated by oral administration of β-eudesmol in the gastrointestinal tract may be transferred to the autonomic center. Extracellular histamine concentration was elevated in the tuberomammillary nucleus of the hypothalamus after oral-ingestion of β-eudesmol. Previous reports have suggested the neural or endocrine system as a possible mechanism to explain the interaction between the hypothalamus and gastrointestinal tract. Orally administered food ingredients affected efferent autonomic nerve activities by neuronal transmissions from the gastrointestinal tract to the central nervous system through the abdominal afferent vagal pathway^32,33^. TRPA1 was reported to be expressed in the enterochromaffin cells of the small intestine^7^, which modulate 5HT_3_ receptor-expressed afferent nerves through secretion of serotonin following TRPA1 activation^34^. Serotonin released by enterochromaffin cells activated extrinsic nerve endings in the gastrointestinal tract, and this may be a trigger for stimulus-induced colonic motility^7^. TRPA1 expression has been reported in autonomic nerves^35^, however, GVNA elevation by β-eudesmol was not eliminated by subdiaphragmatic vagotomy, suggesting that abdominal afferent vagal pathways may not be involved in the communication between the gastrointestinal tract and central nervous system in β-eudesmol derived GVNA elevation. Histamine H_3_ receptor ligands have been suggested to control appetite^36^. Our results suggested β-eudesmol had non-direct effects on histamine H_3_ receptors, since β-eudesmol did not activate histamine H_3_ receptors *in vitro*. Further studies are required to accurately understand the relationship between β-eudesmol derived autonomic nerve effects through TRPA1 and histamine H_3_ receptors. It is important to note that a direct effect of β-eudesmol on the central nervous systems should not be excluded by our experiments, even though β-eudesmol did not directly activate histamine H_3_ receptors in our *in vitro* study.

Emery *et al*. reported that oral-administration (15 mg/kg) of AITC, a TRPA1 activator, increased GLP-1^37^. β-Eudesmol also activates TRPA1, however, it is a weaker activator than AITC^11^. Oral administration of β-eudesmol did not significantly increase GLP-1 in our experiment (control group, 6.21 ± 0.52 pg/ml; β-eudesmol group, 7.31 ± 0.37 pg/ml), however, it was used at a lower dose (1.7 ng/kg) in this study. Our findings suggested an alternative significance of the TRPA1 channel, in addition to the previously reported effects on the endocrine system.

A recent study demonstrated that another representative TRPA1 activator, cinnamaldehyde, suppressed food intake and ghrelin secretion in mice^38^, however, our results showed that oral administration of a representative TRPA1 activator elevated GVNA and food intake increment in rats. This discrepancy may be due to the difference in administration concentration of TRPA1 activators. As reported previously, high dose administration of the TRPA1 activator cinnamaldehyde, which may fully activate TRPA1, was recognized as a noxious stimulus, hence it induced suppression of food intake and ghrelin secretion to avoid noxious foods. The TRPA1 activator β-eudesmol was used at a lower dose in our *in vivo* study, which was almost the same concentration as in beer. In addition, β-eudesmol was reported to be a weak activator of TRPA1^11^. The appetite enhancing effects of cinnamaldehyde have been reported to occur by inhalation^39^. A lower dose (50 ppb) of orally administered cinnamaldehyde in rats produced a slight elevation in mean GVNA in our experiments (control group, 83.7 ± 1.42%; β-eudesmol group, 89.9 ± 2.01%: p < 0.05). Thus, the data suggested that an appropriate dose of TRPA1 activator is expected to act as an appetite stimulant. In humans, appropriate utilization of spices and seasoners in foods and beverages are considered to stimulate the appetite whereas excess amounts are considered to be noxious.

Our results suggested that TRPA1 is involved in stimulative appetite control by β-eudesmol, however, it is important to note that our results do not exclude other target molecules. β-Eudesmol has been reported to stimulate gastric emptying and small intestinal motility by inhibition of the dopamine D_2_ receptor and 5HT_3_ receptor^40^. Previous reports suggested other TRP channel members, such as TRPV1, TRPV2 and TRPM8, also contribute to digestive tract motility^41,42^, however, β-eudesmol did not activate these targets in *in vitro* experiments^11^. In addition to the efferent autonomic nerves, controlled by the central nervous system, the gastrointestinal tract has a local reflex system which includes interneurons, excitatory and inhibitory motor neurons, and smooth muscle which all play significant roles in modulating movement^43,44^. The effects of β-eudesmol on local reflex systems of the gastrointestinal tract need to be clarified. To understand the effects of β-eudesmol on the whole mechanism of appetite promotion, further comprehensive studies are required. Our results revealed a new physiological role for TRPA1 on appetite stimulation through efferent autonomic nerve activity, which will lead to new application possibilities of TRPA1 activators in foods and beverages.

## Materials and Methods

### Materials

β-Eudesmol was purchased from Wako Pure Chemical Industries (Osaka, Japan). Thioperamide and carboxymethylcellulose were purchased from Sigma-Aldrich (MO, USA) and Calbiochem (MA, USA). Rat TRPA1 channel expression vector was purchased from OriGene Technologies (MD, USA).

### Animals and diets

Wistar rats were housed under a 12 h light/12 h dark cycle in a temperature- and humidity-controlled room. Rats were adapted to new housing conditions for at least one week, and healthy rats were used for experiments. Food (type MF or CE2; Oriental Yeast Co., Tokyo) and water were freely available. To monitor feeding, we used a fixed feeder cage for power bait (110 × 60 mm, KN-675-4, Nazme, Tokyo, Japan) with power bait (CE-2). To monitor drinking, we used the KN-670 Nazme water bottle (250 ml, Nazme). We measured the intake of food and drinking water daily by weighing the feeder cage and water bottle. The normalization interval used to calculate the average experimental period was 24 hours. The study was conducted in accordance with the guidelines for animal care, handling, and termination from Kirin Company (Approval Number: YO12-00124, YO14-00175, AN10398-Z00), National Institutes of Natural Sciences (Approval Number: 15A162), and ANBAS Corporation (Approval Number: ANBAS00206, ANBAS00212, ANBAS00248, ANBAS00253, ANBAS00256, ANBAS00258, ANBAS00280, ANBAS00284, ANBAS00291, ANBAS00300, ANBAS00340, ANBAS00354, ANBAS00398, ANBAS00416, ANBAS00475), which are in line with international and Japanese guidelines of animal care and welfare.

### Measurement of ghrelin and GLP-1 levels in rat plasma

Plasma was collected from the jugular vein under anesthesia (1 g/kg urethane, intraperitoneal (i.p.)). Plasma samples were immediately frozen in liquid nitrogen and stored at −80 °C until use. Octanoyl ghrelin and desacyl ghrelin were measured using the Active Ghrelin ELISA Kit (LSI Medience Corporation, Tokyo, Japan) and Desacyl Ghrelin ELISA Kit (LSI Medience Corporation), respectively. In accordance with the manufacturer’s instructions, EDTA blood collection tubes containing aprotinin (361017, Becton, Dickinson and Company, Tokyo, Japan) were used to avoid degradation of octanoyl ghrelin. GLP-1 was measured using the GLP-1 Active form Assay Kit-IBL (Immuno-Biological Laboratories Co., Ltd., Gunma, Japan). To avoid degradation of GLP-1, diprotin-A (Bachem, Torrance, CA, USA), an inhibitor of dipeptidyl aminopeptidase IV, was added to the blood collection tubes in accordance with the manufacturer’s instruction.

### Microdialysis

Microdialysis experiments were carried out on anesthetized rats. Briefly, the rats were anesthetized with isoflurane using a Univentor 400 anesthesia unit (AgnThos, Lidingö, Sweden) and placed in a stereotaxic frame (David Kopf Instruments, Tujunga, CA, USA) using a flat skull position with incisor bar set to − 3.2 mm. The body temperature of the rat was controlled by a rectal thermometer and maintained at 37 °C using a CMA/150 temperature controller (CMA/Microdialysis, Stockholm, Sweden). One hole for the guide cannula and three holes for the anchor screws were drilled using a fine trephine drill. The guide cannula for a microdialysis probe (Eicom Corp., Kyoto, Japan) was implanted into the TMN at the coordinates AP − 4.3 mm; L + 1.1 mm; V − 7.1 mm (from bregma and the dural surface). The whole assembly was secured with dental cement (Dentalon Plus, Heraeus, Germany) and the rats were allowed to recover for 5 days. Rats were fasted from the night before the experiment. On the day of the experiment, the microdialysis probe (0.22 mm o.d., 2 mm membrane length with 50,000 Da cut-off, Eicom CX-I, Eicom Corp.) was inserted into the guide cannula of the anesthetized rat. The rat was then anesthetized by intraperitoneal administration of 10% w/v urethane (1 ml/100 g body weight). The body temperature of the rat was controlled by a rectal thermometer and maintained at 37 °C during the experiment. The inlet and outlet tubing were connected to a 1 ml syringe mounted in a CMA/102 microinjection pump (CMA/Microdialysis) and an Eicom fraction collector (Eicom Corp.). The probe was perfused with artificial CSF solution (148 mM NaCl, 4 mM KCl, 0.8 mM MgCl_2_, 1.4 mM CaCl_2_, 1.2 mM Na_2_HPO_4_, 0.3 mM NaH_2_PO_4_, pH7.2) at a flow-rate of 1 μl/min. Following a 2 hour stabilization period, the samples were collected every 20 min using an Eicom fraction collector. The first 4 samples were taken for determination of basal extracellular levels of HA. Thereafter, the test compound or vehicle was administered p.o. through a gavage from 1:00 to 2:00 pm and the fractions were collected for an additional 180 min. The gastric cannula was intubated into the rat stomach 5 min before administration of the test compound or vehicle. After administration, the gastric cannula was kept in place until the end of sample collection. Concentrations of histamine in the brain microdialysis samples were determined by high performance liquid chromatography (HPLC) with fluorescence detection. The HPLC system included three isocratic HPLC pumps each with an inbuilt degasser unit, a temperature oven, a CMA/200 refrigerated microsampler (CMA/Microdialysis), and a fluorescence detector L-7480 (Merck/Hitachi, Ibaragi, Japan). The fluorescence detector was equipped with a 12 μl flow cell and operated at an excitation wavelength of 340 nm and an emission wavelength of 450 nm. The chromatograms were recorded and integrated using a Clarity computerized data acquisition system (DataApex, Prague, Czech Republic). A HPLC column EICOMPAK SC-5ODS (3.0 mm, i.d. × 150 mm) and a precolumn PREPAKSET CA-ODS (3.0 mm, i.d. × 4 mm) were purchased from Eicom (Kyoto, Japan). The mobile phase (pump A) was a mixture of 0.1 M NaH_2_PO_4_ buffer, methanol (9: 1 v/v) and sodium 1-octanesulfonate, at a final concentration of 0.786 mM; the flow-rate was 500 μl/min. The *o*-phthalaldehyde (OPA) solution (pump B) was prepared using methanol and water to a final concentration of 0.596 mM OPA; the flow-rate was 100 μl/min. Potassium carbonate solution (0.5 M) was supplied by pump C at a flow-rate of 100 μl/min.

### Histamine H_3_ receptor assay

The histamine H_3_ receptor assay was performed using PathHunter^TM^ eXpress β-arrestin GPCR assay kit (DiscorRx Corporation, CA), according to the manufacturer’s instructions.

### Generation of the TRPA1 knockout rats

ZFN constructs specific for the rat TRPA1 gene to delete exons 22 to 24 were designed, assembled, and validated by SAGE LABS (PA, USA). Selected ZFNs were targeted to introns 21 and 24 of the TRPA1 gene, which encodes the ion channel pore (target sequence at intron 21; TACTACCCCACTTTAatcttcGTGGCTGTTTGTAGTA: intron 24; CTCCCTTGTCACATTgtgcaCATGCTGTACATTAG). ZFN mRNAs were microinjected into single-cell rat embryos. Injected embryos were transferred to the oviduct of day 0.5 pseudopregnant rats. Offspring were screened for the ZFN-induced deletion at the target site of the TRPA1 gene. For initial screening, genomic DNA was purified from tail-tip biopsies using QuickExtract™ DNA Extraction Solution (Epicentre). PCR was performed on the purified DNA samples using multiple sets of primers (intron 21 forward primer; TCACCTCCTAAGAGTTGCTGG, intron 21 reverse primer; TAGCCATGTTCACAAAGGCA: intron 24 forward primer; TGGGACTGTGTGGTGCTTTA, intron 24 reverse primer; AGCCAAAGATAGGCCATTGA) designed from the length of nucleotide sequences flanking the ZFN site. PCR products were identified by agarose gel electrophoresis and ethidium bromide staining and the sites of mutations resolved by DNA sequence analysis. Attempts were made to mate a founder with wild-type rats. Genotyping of offspring was performed on genomic DNA extracted from tail-tip biopsies and genomic PCR with specific primers (Mt-F; CCTGATTTGCTCTGGCACA, Int24CellF; TGGGACTGTGTGGTGCT, Int24IgdelR; CCACAGTTCACCACCTTTCA). TRPA1 mutant heterozygotes were intercrossed, and phenotypic characterization was performed on wild-type and homozygous mutant offspring. Preparations of primary rat dorsal root ganglion neurons and calcium imaging were performed according to a previous report^45^.

### Whole-cell patch-clamp experiments

HEK293 cells were maintained in Dulbecco’s modified Eagle’s medium (supplemented with 10% fetal bovine serum, penicillin, streptomycin and L-glutamine and transfected with expression vector using Lipofectamine™ LTX (Life Technologies). The cells were transfected with rat TRPA1 and 0.1 µg pGreen Lantern-1, and incubated with Dulbecco’s modified Eagle’s medium (supplemented with 10% fetal bovine serum, penicillin, streptomycin and L-glutamine) for 14 to 24 hours. The bath solutions for the patch-clamp experiments contained 140 mM NaCl, 5 mM KCl, 2 mM MgCl_2_, 2 mM CaCl_2_, 10 mM glucose, 10 mM 4-(2-hydroxyethyl)-1-piperazineethanesulphonic acid (HEPES), pH 7.4 (with NaOH). Calcium-free bath solutions for patch-clamp experiments contained 140 mM NaCl, 5 mM KCl, 2 mM MgCl_2_, 10 mM glucose, 5 mM ethylene glycol tetraacetic acid (EGTA), 10 mM HEPES, pH 7.4 (with NaOH). The pipette solution contained 140 mM KCl, 5 mM EGTA, and 10 mM HEPES, pH7.4 (with KOH). Data from whole-cell voltage-clamp recordings were sampled at 10 kHz and filtered at 5 kHz for analysis (Axon 200B amplifier with pCLAMP software, Axon Instruments). The cell was voltage-clamped at -60 mV. The current-voltage relationship was obtained using 500 ms voltage-ramp pulses from -100 to + 100 mV, applied every 5 sec. All experiments were performed at room temperature, except for eudesmol solution preparation described above.

### *In vivo* measurements of autonomic nerve activities

Under anesthesia (1 g/kg urethane, intraperitoneal (i.p.)), a polyethylene catheter was inserted into the left femoral vein for intravenous (i.v.) injection. The depth of anesthesia was monitored by the paw pinch method, as described previously^18^. Rats were then cannulated intratracheally, and fixed in stereotaxic apparatus while body temperature was maintained at 37.0–37.5 °C using a heating pad. To record GVNA, a rat was fixed, facing upward, to stereotaxic apparatus, a branch of the ventral subdiaphragmatic vagal nerve on the esophagus was then identified and exposed after incision of the abdomen midline^46^. The distal end of the respective nerve was ligated and connected to a pair of silver wire electrodes to record efferent nerve activity. After subdiaphragmatic vagotomy, a projection fiber of the gastric vagal nerve to the stomach was used to record GVNA. The recording electrodes were immersed in a pool of liquid paraffin oil or a mixture of warm Vaseline and liquid paraffin oil to prevent dehydration and for electrical insulation, respectively. The rat was allowed to stabilize for 30–60 min after the nerves were placed on the recording electrodes. Electrical changes in GVNA were amplified, filtered, monitored on an oscilloscope and converted to standard pulses by a window discriminator. The counted discharge rate was sampled with a Power-Lab analog-to-digital converter, and stored on hard disk for off-line analysis. The nerve activity data were analyzed using an average firing frequency value (pulses/5 seconds) every 5 minutes: the normalization interval to calculate the average experimental period was 5 minutes.

### Statistical analysis

Statistical differences were analyzed by the Mann-Whitney U test for comparison between two groups, and the Kruskal Wallis test followed by Steel-Dwass for comparison of multiple groups. P values < 0.05 were considered statistically significant.
